# Midwives' Experiences in Providing Care and Counselling to Women with Female Genital Mutilation (FGM) Related Problems

**DOI:** 10.1155/2013/785148

**Published:** 2013-09-18

**Authors:** Elisabeth Isman, Amina Mahmoud Warsame, Annika Johansson, Sarah Fried, Vanja Berggren

**Affiliations:** ^1^Department of Public Health Sciences, Karolinska Institutet, 171 77 Stockholm, Sweden; ^2^Development Studies, Hargeisa, Somaliland, Somalia; ^3^Department of Health Sciences, Faculty of Medicine, Lund University, 221 00 Lund, Sweden

## Abstract

*Aim*. The aim of this study was to elucidate midwives experiences in providing care and counselling to women with FGM related problems. *Setting*. The study was conducted at a maternity clinic in Hargeisa, Somaliland. *Method*. A qualitative, inductive study were performed with eight midwives living in Somaliland. The interviews had semi-structured questions. Content analysis was used for the analysis. *Findings*. The main findings of the present study were how midwives are challenged by culture and religion when providing FGM counselling. The most prominent challenge is the perception that FGM is an important part of the culture and from this point of view the midwives work is apprehended as interfering and subverting the Somali culture. Having personal experience of FGM emerged as a benefit when counselling women. *Conclusion*. There is a contradiction between the professional actions of performing FGM despite a personal belief against FGM. Midwives as a professional group could be important agents of change and further research is needed about the midwives role in this process.

## 1. Introduction

Female genital mutilation (FGM) is currently practiced in 30 African countries in the sub-Saharan and northeastern regions and in some countries in Asia and in the Middle East [1]. 

FGM is a serious public health problem and a global concern. It is estimated that around 140 million girls and women worldwide have undergone FGM and that at least two million girls are annually at risk of undergoing some form of the procedure. FGM is interfering with one of the most intimate aspects of a woman's life and is a fundamental violation of women's human rights [1].

The age when the primary FGM is performed varies depending on ethnic groups and geographic location. In Somalia and Sudan, FGM is often performed at the age of 5–10 years. In other areas, the procedure is undertaken on baby girls (Yemen) or at puberty (Sierra Leone) [2–4]. The motives of FGM are complex and vary between different contexts and are surrounded with myths and taboos [5]. Although the origin of FGM is concealed, the practice survives today and it is a culturally embedded phenomenon. Among those who practice it, FGM is often performed due to tradition, cultural conformity, or and religious reasons. It is thought to hamper women's sexuality and preserve honor and virginity, thus enhancing their marriageability and marking ethnic boundaries [1, 6–9].

Since FGM practices differ greatly between ethnic groups and geographic regions, WHO has classified the predominant types of mutilation into four categories. *Type I, clitoridectomy*, with partial or total removal of the clitoris or only the prepuce (the fold of skin surrounding the clitoris); *Type II, excision*, with partial or total removal of the clitoris and the labia minora, with or without excision of the labia majora; *Type III, infibulation*, with narrowing of the vaginal opening through the creation of a covering seal. The seal is formed by cutting and repositioning the inner, or outer, labia; *Type IV*, *other* comprises all other harmful procedures to the female genitalia for non-medical purposes, for example, pricking, piercing, incising, scraping and cauterizing the genital area [10].

The categories are helpful in the effort to bring uniformity to the research on FGM. Types I and II are the most common forms, accounting for approximately 80%, while infibulation (Type III) is found around 15% of the cases worldwide [10]. All types of FGM have immediate and long-term health consequences depending on the type performed, the expertise of the circumciser, the hygienic conditions under which it is performed, and the amount of resistance and general health condition of the girl/woman undergoing the procedure [9, 11–13]. The health consequences are numerous, ranging from severe pain and bleeding to risk of infection which may result in shock and death. Most complications are reported in relation to the most severe form of FGM, infibulation [14–17].

The highest prevalence of FGM has been reported in Somalia and Djibouti where 98% of all women have undergone some form of the procedure [18–21]. In Sudan, Somalia, and Eritrea, the most severe form, infibulation, has the highest prevalence [20–23]. FGM is a deeply rooted tradition in the Somali culture in Somaliland, and it is a difficult practice to monitor due to its close link to culture and tradition and because it concerns an intimate aspect of women's life. FGM is deeply embedded in society, and its elimination requires a clear understanding of the cultural perceptions and beliefs it feeds on. Old traditions as FGM are hard to discard especially when they are culturally rooted. However, recent years awareness campaigns against the practice seem to have resulted in that more Somalis are questioning the tradition of infibulation [13].

In 2009, Somalia had a maternal mortality of 1.200 per 100.000 live births. In a report from UNFPA (2011), Somalia was mentioned as one country who needs to dramatically scale up the workforce of midwives to be able to reduce the maternal mortality. Midwives are a core group of professionals who meet women in different stages and needs during their reproductive age. International studies have looked upon ethical dilemmas among health care providers working in reproductive health services. FGM has been recognized to create difficult challenges from an ethical and legal perspective [24, 25]. Placed between the requests from families for performing FGM on the little daughters and the knowledge that FGM may cause lifelong harm to their health, midwives may find themselves in difficult, ethical dilemmas [26]. The aim of this study was to elucidate midwives' experiences in providing care and counselling to women with FGM related problems.

## 2. Study Setting

The network against Female Genital Mutilation in Somaliland (NAFIS) is an umbrella nongovernment organization (NGO) and consists of 19 local NGOs from the different regions of Somaliland. NAFIS aims to minimize the consequences of and eventually eradicate all kinds of FGM from Somaliland. Since its establishment in 2006, NAFIS has worked with policy advocacy, sensitization workshops, training programs, research, and awareness raising campaign about the harmful effects of FGM. In 2011, a Support Center was established by NAFIS in the capital Hargeisa to meet the need for maternity clinic in the outskirts of the town and serve a poor area in an IDP (internally displaced people) camp, mainly consisting of minority Somalis. Somaliland is a self-declared republic in northern Somalia at the horn of Africa. It has its own democratically elected government and public institutions and has witnessed relative stability for the last two decades. It is not internationally recognized.

Parallel with the services provided at the Support Center, NAFIS organizes workshops and community dialogues in the catchment area of the Support Center. In meetings with local women's groups, religious leaders, village health committees, and other local institutions, NAFIS informs about the health consequences of FGM and initiates dialogues on FGM in Islam, on the rights of girls and women for physical integrity, and on the changing cultural and social context of FGM in their own communities.

### 2.1. Study Design

Due to the scarcity of studies on FGM in Somaliland and to the delicate and intimate nature of the problems dealt with, we chose an exploratory qualitative research design, based on interviews with midwives. The criteria for selecting midwives for the interviews were that they had training on and experience of care and counselling of women with FGM related problems. Using convenience sampling, we selected all five midwives who worked at the Support Center and were trained by NAFIS in FGM related care and counselling. Three additional midwives who had received the same training but worked in another clinic in Hargeisa were also asked to participate. The midwives were briefed on the purpose of the study and assured anonymity. They were told that they could decline or interrupt the interview at any moment. None declined and all completed the interview. The five-member research team representing nursing/midwifery and social sciences from Somaliland and Sweden together designed an interview guide which consisted of semistructured questions covering the main areas that we wanted to cover and some open questions (Appendix B). One of the research team members, a trained female Somali social scientist with long experience of qualitative interviewing and deep knowledge of FGM issues (Amina Mahmoud Warsame), conducted a pilot study including three interviews to explore issues of particular importance to the midwives. Questions concerning the “turning point,” that is, why and when they had changed their mind regarding FGM, emerged as one of such issues. Another was the potential contradiction felt by the midwives when counselling women who were positive towards FGM. The interview guide was then revised to include the themes emerging from the pilot study. 

The eight interviews were conducted by Amina Mahmoud Warsame in Somali. Each interview lasted around one hour. Saturation was found after interviews with six midwives; another two interviews were performed to confirm the saturation. The interviews were tape-recorded with the permission of the midwives, transcribed verbatim, and translated into English. They were coded to ensure confidentiality [27]. Two trained Somali research assistants made the translations which were then checked by Amina Mahmoud Warsame who is fluent in English. The true meaning of some concepts and expressions were difficult to translate and are therefore presented in the text in Somali (italics) and given an approximate English translation.

### 2.2. Data Analysis

The findings were analyzed using qualitative content analysis [28]. The complete transcribed narratives were first read independently by all team members to obtain a sense of the whole. The material was then assigned meaningful units, coded, and discussed thoroughly within the team to reach agreement on patterns emerging in the narratives and enhance trustworthiness. A number of subthemes were identified and were brought together under three main themes, each one referring to a descriptive level of content and thus expressions of the manifest content of the text. In the presentation of findings, we follow the midwives terminology. Thus, sunna and circumcision/pharaonic are used instead of mutilation. To enhance both trustworthiness and credibility, each theme is enriched with quotations to remain close to the narratives [29].

## 3. Background Characteristics

See Table 1.

## 4. Findings

### 4.1. Midwives' Views on FGM and Plans for Own Daughters

Three of the midwives were married and had children; the others were unmarried. Seven of them were infibulated, while one, a 24-year-old unmarried woman, had had sunna performed. All midwives felt that infibulation was a harmful practice, describing it as awful, evil, painful, and terrible. Ambivalent feelings towards having their daughters undergo FGM emerged. Only one of the midwives said clearly that she would “do nothing” to her daughter. Most of the others considered performing sunna, “a mild sunna,” only piercing, or “make it bleed” (Table 1).

Their midwifery experience ranged between around one year and up to 20 years. All midwives had been trained in giving care and counselling to women with problems related to FGM. They all felt confident in giving counselling but expressed a need to have more training.

Analyzing the midwives' narratives of their work with care and counselling of women at the clinic, three main themes emerged: (1) midwives' encounters when providing FGM counselling, (2) benefits of own FGM experiences when counselling women, and (3) midwives' views on challenges and problems in future anti-FGM works. The themes were building on subthemes which sometimes were overlapping (Table 2).

### 4.2. Midwives' Encounters When Providing FGM Counselling

#### 4.2.1. Midwives Feeling Confident and Supporting

The counselling provided was given both in groups and individually, depending on the needs and condition of the women. All midwives felt confident in giving counselling related to FGM. They described however that they would like to increase their skills through further studies and training in public health to be able to deal with new knowledge and development in the field of FGM. The confidence the midwives expressed was enhanced by the perception that the society views their work as honorable and beneficiary. In particulary people who had gained knowledge on harmful health consequences associated with FGM considered the work of the midwives as positive, and the midwives felt that they were supported by this group.

*“Many people who understand the problems of FGM see our work as a good service, as they acknowledge its problem and the pain during the circumcision, at the time of the wedding and during child birth”  (Midwife 47 years)*

#### 4.2.2. Midwives Being Challenged by Culture and Religion

The obstacles the midwives faced in their work of giving care and counselling to women experiencing FGM related health problems were closely linked to cultural beliefs, tradition, and religion. The most prominent obstacles appeared to be connected with the perception that FGM is an important part of the culture. The midwives had been told that they were interfering in family affairs and subverting the Somali culture. According to the midwives, it is women who are unaware of the health consequences of FGM who believe that midwives working with counselling are attacking their culture. Parents stated that FGM is their culture and that they want their daughters to continue the practice.

Both culture and religion were mentioned as arguments against FGM counselling. That abandoning FGM will cause Allah's anger was a belief the midwives could meet in their work with counselling, expressed as “*caado la gooyaa, cadho Allay leedahay*” (literally meaning that abandoning a tradition can cause God's anger). A major challenge that the midwives encountered was women having “superstitious beliefs” about FGM.

*“Some women simply say that they will do the same type to their daughters without knowing the consequences. Some mothers say that they will never expose their daughters to the practice while there are others who say that if girls are spared from infibulation and set free without preventive stitches, how can they be protected?”*  *(Midwife 47 years)*

#### 4.2.3. Women's Delay in Seeking Health Care

It was an overall impression emerging from the midwives' narratives that most women endure and hide their health complications for a rather long time. Reasons given behind this were lack of money, lack of knowledge, and/or being shy to talk about their problems. Women were said to feel ashamed of publicly confessing their problem. The midwives described how it often requires lengthy explanations and counselling regarding FGM and its health consequences before a woman can tell the truth of her problem. Some women were said not to come to the clinic because they are afraid or they do not want their problems to be known. 

To be able to provide assistance for women seeking care, the midwives pointed out the importance to understand the women's perception and knowledge and by so building their confidence and gaining their trust. According to the midwives, it is the poorest women who suffer the brunt of the problems related to FGM; they mostly hide the pain as a result of no access to doctors and lack of money to pay the bill or to buy medicine. Some midwives had met women suffering from their problems between 10 to 30 years without seeking care.

*“As a rule the women do not seek help as soon as they experience the problems”  (Midwife 28 years)*

One midwife with more than 20 years of working experience said

* “They think that the problem is unique to them”  (Midwife 47 years)*

*“Both of them were hiding it until the time that they are giving birth and they could not hide it any more”*  *(Midwife 39 years)*

### 4.3. Benefits of Own FGM Experience When Counselling Women

#### 4.3.1. Sharing a Common Experience

The midwives that had undergone infibulation had the procedure done at a young age. In their present work, they all perceived this experience as positive. They felt accepted and felt that the patients were listening to them knowing that they have had the same experience, both from procedure and often from personal experiences of FGM related problems. A strength of being circumcised yourself was that they could relate to the women and understand their problems since they have the same “cutting of the genitals.”

*“Yes, I tell the women that I am like them and I understand their problems. Since some of the women are very shy, they feel confident when I tell them that”  (Midwife 42 years)*

*“We are in the same boat. The women can also guess that I am circumcised in the same way as them and perhaps that can be positive in the sense that deep down they know I can understand what they are feeling and relate to them better”  (Midwife 29 years)*

#### 4.3.2. Changing Opinions regarding FGM

Personal experience, health education, and listening to religious leaders are reasons given by the midwives as to why they have now rejected and abandoned infibulation. Most of the midwives described that they have gone through a process when changing their minds regarding FGM. They described different reasons and experiences that had led to the moment where a change of mind had occurred. Religion and health consequences were repeatedly mentioned as reasons. Their reasons were also based on their own experience of physical and psychological suffering related to their FGM and consistent with an understanding that FGM is not based on Islam. Most of the midwives expressed opinions concerning circumcision/infibulation as being an awful, evil, and harmful practice with no benefits. 

Concerning their own circumcision, it was regarded as something that has already happened and could not be undone. They concluded that focus now should be to save girls from the same fate. For example, one midwife explained how she was first happy about the upcoming event of being circumcised but how after the circumcision she had urine retention and the stitches tore open resulting in a second infibulation. She suffered from recurrent infections and painful wedding night and now perceives FGM as harmful.

A newly educated midwife said

*“Religion does not allow anything to be cut. There is no xaaraan (unclean) flesh in the body”  (Midwife 39 years)*

*“I see circumcision as something with many painful difficulties. It is associated with pain and problems such as painful menses, cutting all the time, when a girl marries and when she is giving birth”  (Midwife 25 years)*

### 4.4. Midwives' Views on Challenges and Problems in Future Anti-FGM Work

#### 4.4.1. Female Decision Makers

When it comes to who decides whether to perform FGM on girls, all midwives state that it is first and foremost the mother in the family. From the midwives' interviews it emerged that their own mothers and grandmothers adhered to the practice of FGM, believing it was a cultural heritage based on religion and that circumcision was perceived as something good for the women. The mother and grandmother in a family propose the idea, and the father pays the practitioner. The fathers could have a say regarding if and what type of FGM to perform, but the mothers exert a strong influence on the decision.

*“It is the mothers who are queens when it comes to taking the decision. Even if the father does not want the daughter to be circumcised the mothers find ways to do it”  (Midwife 39 years)*

*“My mother hid my circumcision from my father and did it secretly as she knew that he disapproves of FGM”  (Midwife 28 years)*

However, if there are different opinions regarding performing FGM, it appears to be the father who takes the final decision. Other members of the family can try to influence the decision, but their impact is minor.

#### 4.4.2. Still Performing FGM in spite of Being Against

A contradiction emerged among the midwives between professional action and personal beliefs towards FGM. The same midwife who was personally against FGM still performed mild forms of FGM on her girl relatives to satisfy her mother and grandmother. Similar stories came from several midwives performing sunna type of circumcision, while at the same time trying to convince their families not to perform infibulation, but rather sunna.

Difficulties in suddenly abandoning the practice were mentioned as one explanation of why they were still performing FGM. Another reason given was the pressure coming from young female relatives who expressed a wish to be infibulated as their friends had been. Ambivalent feelings towards having their daughters undergo FGM emerged. Among the midwives' daughters, it ranged from some who already had sunna performed to either perform sunna or piercing of the clitoris, or maybe to leave them untouched. Nevertheless, the midwives expressed a belief that each generation will be better than the preceding one and that even sunna will vanish with time.

*“I did a very mild type for my nieces”  (Midwife 25 years)*

*“Small girls like to do what their peers are doing and FGM is not an exception”  (Midwife 47 years)*

*“I do not like to change Allah's creation”  (Midwife 28 years)*

#### 4.4.3. Importance to Reach Out with Information about Virginity and Religion

One crucial factor that emerged from the midwives' narrations regarding the abolition of FGM was the importance to reach out with the message that virginity is not synonymous with infibulation and that infibulation is not virginity. Some of the midwives stated that virginity is “God made.” To achieve abolition of FGM and make it sustainable, religious leaders have to be involved in information campaign regarding FGM, declaring that it should be totally abandoned. Many people get together for Friday prayers and this was mentioned as a good opportunity to spread information. 

Other important interventions described were visiting schools to inform young girls about the harmful effects of FGM. Housewives that mostly stay at home and have little social interaction can be reached by house-to-house awareness campaigns. The midwives stressed the importance to reach those who still accept FGM and are unaware of the lifelong complications that follow the procedure. It emerged from the midwives that previously it was taboo to discuss FGM but, after improved education and awareness campaigns by civil society organizations, this has changed. As a result, there are even some practitioners who now refuse to perform infibulation even though they are being offered money to do it. However, the midwives emphasized that there are still possibilities to have infibulation done by going to an old lady who never had awareness raising or information on the issue. 

Almost all midwives talked about the need of interventions to achieve a sustainable change in FGM practices through providing the practitioners with alternative jobs and income. Other means mentioned were distribution of printed stickers with cultural and religious slogans to be posted on walls along main roads and other popular places. Some midwives suggested having a police unit to follow up the practitioners and advice people to report those who perform it.

*“Intensive information must be disseminated on the issue of virginity in the sense that virginity is God made and not synonymous with the stitching of the female organ”  (Midwife 42 years)*

*“First it is women who hold the key to ending the practice”  (Midwife 24 years)*

*“Traditional birth attendants should be stationed in mother and child centers to convince people that they have abandoned the practice”  (Midwife 28 years)*

*“For FGM to stop there is also a need for alternative employment to old women who do pharaonic circumcision”  (Midwife 47 years)*

## 5. Discussion

The aim of this study was to elucidate Somali midwives' experiences in providing care and counselling to women with FGM related problems. This study revealed that the midwives are challenged by both culture and religion in their work with FGM related counselling. The most prominent challenge is the perception that FGM is an important part of the culture, and, from this point of view, the midwives' work is apprehended as interfering with and subverting the Somali culture. A belief that abandoning FGM will cause Allah's anger also emerged as a challenge for the midwives. A clear message from the midwives was the importance to reach out with information regarding virginity and religion. Virginity is “God made” and not synonymous with infibulation; this message was seen as vital in order to reach abolition of FGM. Another important step to make abolition sustainable was that religious leaders have to be involved in the awareness campaigns. Religious leaders have a strong position in the society and can therefore have a great impact on changing traditions such as FGM. 

Despite the challenges, all midwives felt confident in giving FGM counselling mainly due to the fact that they also felt appreciated by many in the society who saw their work as honorable.

However, the midwives in this study expressed a need to have a deeper knowledge to perform counselling. Strengthening the competence of the midwives has been recognized internationally to improve the reproductive health care for women. According to Miller et al. [30], training health care providers on communication skills is one way to improve the quality of care and counselling. Further training will encourage and empower midwives and give them useful tools in their work with counselling to and care of women affected by FGM. Together with awareness campaigns, this can increase the impact of improved skills and help to change the perception of the community on both health implications and myths associated with FGM. 

Several different ways to conduct campaigns were mentioned, such as going to schools to inform girls about the harmful consequences, door-to-door education to reach housewives, and the use of media. Our results from this study clearly indicated that the mothers had the power to take the decision regarding FGM. The midwives described the mothers as queens in taking decision and also consequently being the key to ending the practice. Another important factor for achievement of sustainable change towards the abolition of FGM is to provide the practitioners with alternative jobs and income. This is in line with the views of Asekun-Olarinmoye and Amusan [31] claiming that practitioners will not stop performing FGM unless they receive an income equivalent to what they now earn from the practice.

Shell-Duncan et al. [32] found in their study in Senegal and Gambia a significant change in the location of circumcision, the degree of celebration, and the age when circumcision occurs, but no change in the type or degree of cutting. In our study, all midwives claimed that they reject infibulation. However, some say that they will circumcise their future daughters and some claim that they will leave them untouched. Difficulties to suddenly abandon the practice of FGM were mentioned as one reason for why some of them were still performing sunna type of circumcision. This ambivalence gives an insight into the complexity of the practice.

In this study, personal experience, health education, and information from religious leaders emerged as the main reasons behind the midwives' change of opinion regarding FGM. They described how the process of changing opinion built on their own negative personal experiences of FGM, consistent with an understanding that FGM is not based on Islam. When evaluating or measuring behavior change in regard to FGM, statements of intention not to cut future daughters are commonly used. Whether these statements show an actual change in behavior or indicate a change in attitude depends on the validity of using public statement as an indicator of personal behavior. Motivation to change is influenced by the balance between perceived benefits and perceived risks. Whether the midwives will show an actual change in behavior towards the circumcision of their daughters, or indicate a change in attitude, remains to be seen. Nevertheless, abandoning infibulation is one step in the direction of abolishing FGM. A crucial stage in the behavioral change process to move away from FGM is the maintenance of a decision [33]. In this stage, midwives have an essential role to support women in having nontraditional perception of FGM and to encourage their endeavors to abandon this harmful tradition.

Based on these findings, more knowledge is necessary concerning how midwives can be supported to provide high physical and psychological care for girls and women suffering from complications of FGM and, not least, how they can resist requests to perform FGM. 

## 6. Limitations of the Study

Several aspects have been taken into consideration to increase the trustworthiness of this study. It might be argued that the interview language could have been a limitation due to the need to translate it into English with possible loss of words and/or change of meaning. However, in order to avoid misunderstanding and to enhance the validity of the translation, the conductor of the interviews read all of the translations, as well as listening to the tapes, to confirm the correctness of the translations. In addition, a strength of having a native speaker conducting the interviews is that it reduces the risk to create a barrier in the contact when interviewing someone about a sensitive issue such as female genital mutilation. A possible information bias should be considered; all interviewed midwives were working with care and counselling of women affected by FGM and trained by NAFIS. Asked about their personal opinion concerning FGM, they might have felt uncomfortable to reveal positive feelings or attitude towards FGM if they had such feelings. This possibility was discussed between the interviewer (Amina Mahmoud Warsame) and the senior midwife at the center, who saw the risk for information bias as very limited.

The small study sample could be seen as a limitation as the findings are not generalizable; however, that is not the aim within qualitative research, but rather it is to explore in depth how people perceive and experience the issues concerned. 

## 7. Conclusion and Recommendations

The conclusion of this study is that FGM is still a challenge to work against due to the strong links to culture, tradition, and religion. Strengthening the midwives' competence in communications skills will enhance their work with care and counselling.

This study reveals an important contradiction between professional actions in performing FGM despite a personal belief against it. A challenge is to support midwives to resist requests for performing FGM. Hence, more research is needed concerning midwives as a professional group, since they could be important agents of change to achieve abolishing of FGM for the abolishment of FGM.

## Figures and Tables

**Table 1:** Background characteristics and views on the eight midwives participating in the study.

Midwife no.	Age	Type of FGM	Civil status	Education	Children	Daughters and FGM	Perception of FGM	Working years as midwife
1	25	Type III	Single	Sec. school Nursing school 3 years	No children	Will not perform FGM or sunna	“Awful, evil practice”	7 years at Magan

2	47	Type III	Married	Sec. school Nursing school 1 year	2 sons, 2 daughters	Sunna done on both daughters at 7 Father advised against	“Harmful practice” (infib)	26 years at Daami

3	24	Sunna	Single	Sec. school Nursing school and midwifery	No	“I will do sunna for her”. Might leave her, or just make bleeding	“I do not like pharaonic circumcision.	3 years at Daami MCH

4	28	Type III	Single	Sec. school Nursing school and midwifery	No	No	“Awful practice” (infib)	10 months at Magan

5	39	Type III	Single	Left school at grade 5. Some midwifery training	No	“I will not do anything to them or have piercing performed of clitoris to make it bleed”	“I have bad memories of it”	Several years at Muhammed MCH

6	25	Type III	Single	Sec. school Midwifery training	No	“I will do sunna on my daughters”	“I see it as one with many painful difficulties”	1 year at Daami MCH

7	42	Type III	Married	Sec. school Nursing school	5 sons, 2 daughters	Yes, sunna on both at the age of 8	“Sunna is almost as being untouched”	20 years at Daami MCH

8	29	Type III	Married	Sec. school University 4 years Midwifery 2 years	1 son	Mild type of sunna, piercing of clitoris, or leave untouched.	“Now I know it has only problems but no benefits” (infib)	2 years at Magan Several years at a different hospital before that

**Table 2:** Data analysis.

Themes	Subthemes
Midwives' encounters when providing FGM counselling	Midwives feeling confident and supported Midwives being challenged by culture and religion Women's delay in seeking care

Benefits of own FGM experience when counselling women	Sharing a common experience Changing opinions regarding FGM

Midwives' views on challenges and problems in future anti-FGM work.	Female decision makers still performing FGM in spite of being against it Importance of reaching out with information about virginity and religion

## Appendices

### A. Definitions and Abbreviations

Counselling: Advice and guidance given to women suffering from physical and psychological problems following their FGM.
Deinfibulation: Splitting up the bridge of skin that covers the vulva after infibulation.
Infibulation: Stitching/narrowing of the vaginal opening.
Labia: Anatomical structures that are part of the female genitals, which surround and protect the clitoris, openings of the vagina, and urethra.
Pharaonic: Local expression for infibulation.
Sunna: Local expression for Types I and II. Arabic word for tradition; sunna refers to the saying and actions of Muhammed.
Turning point: Referring to the moment when one changes his/her mind on a subject for various reasons.
FGM: Female genital mutilation.
FIGO: International Federation of Gynaecology and Obstetrics.
GDP: Gross domestic products.
Hadiths: Collections of narratives regarding Muhammed.
ICM: International Confederation of Midwives.
MCH: Mother and child health.
NAFIS: Network against Female Genital Mutilation In Somaliland.
NGO: Nongovernmental organization.
SRH: Sexual and reproductive health.
WHO: World Health Organization.

### B. Interview Guide

The aim of this study was to elucidate midwives' experiences in providing care and counselling to women with FGM related problems.

*Background Demographic Information*

-  Age.
-  Married/unmarried.
-  Number of children; son/daughter; age of the child.
-  If having daughters, have they undergone FGM? What type, at what age, and have all daughters undergone the same form of FGM?
-  If daughters have not undergone the same form of FGM what is the reason behind that?
-  If daughters are untouched what is the motive behind that?
-  How many years have you been working at Magan clinic?
-  What kind of education/midwife training (no. of years, etc.) and how many years of experience in the profession?

*Specific Questions*

(1) How do the midwives look upon their work with care and counselling to women having undergone FGM?   
Please, probe into.
  (i) What are the main obstacles in giving care and counselling?
  (ii) Can it make a difference to be circumcised (or not circumcised) yourself when helping women with FGM related problems?
  (iii) Can it be positive to be circumcised yourself when helping women with FGM related problems?
  (iv) Do you feel that you can give adequate help to the women?
  (v) How do they look upon their own circumcision?
  (vi) Circumcised, if yes, what type and at what age was it done?
  (vii) Uncircumcised, if yes, reasons behind that and what are their feelings behind that?
  (viii) How are your work and position, working with FGM, perceived in the community?
(2) How do the midwives perceive that they could improve their work with care and counselling to women seeking care for FGM related consequences?    
Please, probe into.
  (i) What do you think needs to be improved or strengthened in the work to help women affected by FGM?
  (ii) What needs to be improved in the ability to provide the women with help according to their experience?
  (iii) Why are women seeking care, and do they come soon after starting to have problems or do they tend to wait?
  (iv) Is it easy for women to seek help for FGM related problems?
  (v) Do they come alone?
  (vi) How do the relatives look upon them seeking help?
(3) How do they perceive the future concerning FGM?
Please, probe into.
  (i) How do their own mothers and grandmothers perceive FGM?
  (ii) What influence their mothers, grandmothers, and mothers in law have when it comes to take a decision on whether or not to perform FGM on a girl/daughter? And what about their husband, father, and brothers?
  (iii) Who takes a decision if there is different opinion on performing FGM or not?
  (iv) How do they (midwives) perceive their own daughters future concerning FGM?
  (v) Motives for continuation? Motives for abandoning the tradition?
  (vi) What is according to you the most important factor to be able to cease FGM?
  (vii) Is there something else, apart from what is done now, that you believe can help parents to abandon the practice of FGM?
